# A synthetic co-culture for bioproduction of ammonia from methane and air

**DOI:** 10.1093/jimb/kuae044

**Published:** 2024-11-18

**Authors:** Anna Morgan Crumbley, Shivani Garg, Jonathan Lin Pan, Ramon Gonzalez

**Affiliations:** Department of Chemical and Biomolecular Engineering, Rice Universit, Houston, USA; Department of Chemical and Biomolecular Engineering, Rice Universit, Houston, USA; Department of Chemical and Biomolecular Engineering, Rice Universit, Houston, USA; Department of Chemical and Biomolecular Engineering, Rice Universit, Houston, USA

**Keywords:** Methane, Ammonia, Co-culture, Bio-reforming, Nitrogen

## Abstract

Fixed nitrogen fertilizers feed 50% of the global population, but most fixed nitrogen production occurs using energy-intensive Haber–Bosch-based chemistry combining nitrogen (N_2_) from air with gaseous hydrogen (H_2_) from methane (CH_4_) at high temperatures and pressures in large-scale facilities sensitive to supply chain disruptions. This work demonstrates the biological transformation of atmospheric N_2_ into ammonia (NH_3_) using CH_4_ as the sole carbon and energy source in a single vessel at ambient pressure and temperature, representing a biological “room-pressure and room-temperature” route to NH_3_ that could ultimately be developed to support compact, remote, NH_3_ production facilities amenable to distributed biomanufacturing. The synthetic microbial co-culture of engineered methanotroph *Methylomicrobium buryatense* (now *Methylotuvimicrobium buryatense*) and diazotroph *Azotobacter vinelandii* converted three CH_4_ molecules to l-lactate (C_3_H_6_O_3_) and powered gaseous N_2_ conversion to NH_3_. The design used division of labor and mutualistic metabolism strategies to address the oxygen sensitivity of nitrogenase and maximize CH_4_ oxidation efficiency. Media pH and salinity were central variables supporting co-cultivation. Carbon concentration heavily influenced NH_3_ production. Smaller-scale NH_3_ production near dispersed, abundant, and renewable CH_4_ sources could reduce disruption risks and capitalize on untapped energy resources.

**One-Sentence Summary:**

Co-culture of engineered microorganisms *Methylomicrobium buryatense* and *Azotobacter vinelandii* facilitated the use of methane gas as a sole carbon feedstock to produce ammonia in an ambient temperature, atmospheric pressure, single-vessel system.

## Graphical Abstract

**Graphical Abstract:**
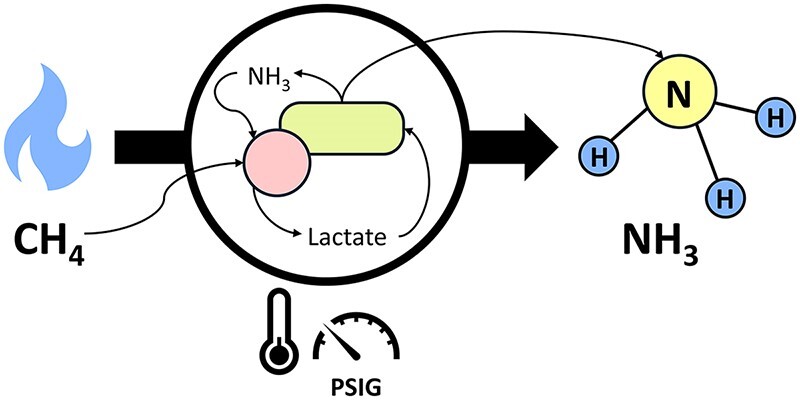

## Introduction

While bioavailable nitrogen was historically a limited agricultural resource, modern chemical fixation of atmospheric nitrogen into ammonia (NH_3_) is a large-scale industry successfully feeding 50% of the world’s population (Erisman et al., 2008). Modern fertilizer production technology is derived from the energy-intensive Haber–Bosch process, which combines nitrogen gas (N_2_) from air with hydrogen gas (H_2_) generated from methane (CH_4_) through steam methane reforming to perform the chemical reaction N_2_ + 3H_2_ → 2NH_3_ over metal catalysts at high temperatures (650–700 K) and pressures (50–200 bar) (Jennings, 1991). However, centralized large-scale NH_3_ manufacturing is increasingly recognized to be susceptible to supply chain disruptions, potentially resulting in inadequate access to chemicals that are essential for foodstuff production (Cooley, 2022). The United States of America (USA) currently contributes approximately 7% of global NH_3_ production (Pattabathula & Richardson, 2016). Developing distributable NH_3_ manufacturing strategies could represent an increased opportunity for supply chain resiliency.

One path to distributable NH_3_ production would be the use of a low-cost, high-energy, renewable feedstock to supply the energy demands of distributed NH_3_ production. Renewable CH_4_ is a high-energy and low-cost one carbon (C_1_) feedstock that represents a globally abundant feedstock for distributed manufacturing (Strong et al., 2015). Sourced primarily from remote landfill, wastewater, and agricultural sites disconnected from established market infrastructure, direct incorporation of remote renewable CH_4_ into modern centralized NH_3_ manufacturing infrastructure is infeasible due to transportation and logistical challenges, since remote renewable CH_4_ is defined, alternatively, both as originating at landfill, wastewater, and agricultural sites distant from established market infrastructure, and being produced at less than 5,000 barrels of oil equivalent (BOE)/day (<29 million ft^3^/day) (U.S. Geological Survey, 2020; Clomburg et al., 2017). Taken together, these remote CH_4_ sources represent almost 240 billion cubic feet of CH_4_ released in the USA annually—an amount of CH_4_ equivalent to 85% of the annual domestic energy required to produce nitrogenous fertilizers (Clomburg et al., 2017; Lorenz, 2018). However, distributing directly scaled-down NH_3_ manufacturing facilities to the remote CH_4_ sources is infeasible due to economy of scale limitations related to the use of expensive catalysts operating at high temperatures and pressures (Garagounis et al., 2019; Reese et al., 2016). Developing an NH_3_ synthesis technology operating at ambient temperature and pressure would open an opportunity to support distributable NH_3_ using remote CH_4_ as the feedstock.

This work demonstrates the biological transformation of atmospheric N_2_ into NH_3_ using CH_4_ as the sole carbon and energy source in a single vessel at ambient pressure and temperature, representing a “room-pressure and room-temperature” route to NH_3_ that could ultimately be developed to support compact, remote, NH_3_ production facilities amenable to distributed biomanufacturing. During the process, multiple energy carriers, including those derived from CH_4_ oxidation by methanotrophic organisms, supply reducing equivalents for biological nitrogen fixation (Ledbetter et al., 2017; Poudel et al., 2018). Biological nitrogen fixation (BNF) then uses an enzymatic process performed by diazotrophic organisms to reduce atmospheric N_2_ in the air (N_2_ + O_2_) and produce NH_3_ (Dixon & Kahn, 2004). Integrating enzymatic CH_4_ consumption and NH_3_ production into a single vessel required establishing synergism of two distinct biological metabolic processes, specifically CH_4_ oxidation and N_2_ fixation.

**Fig. 1.**
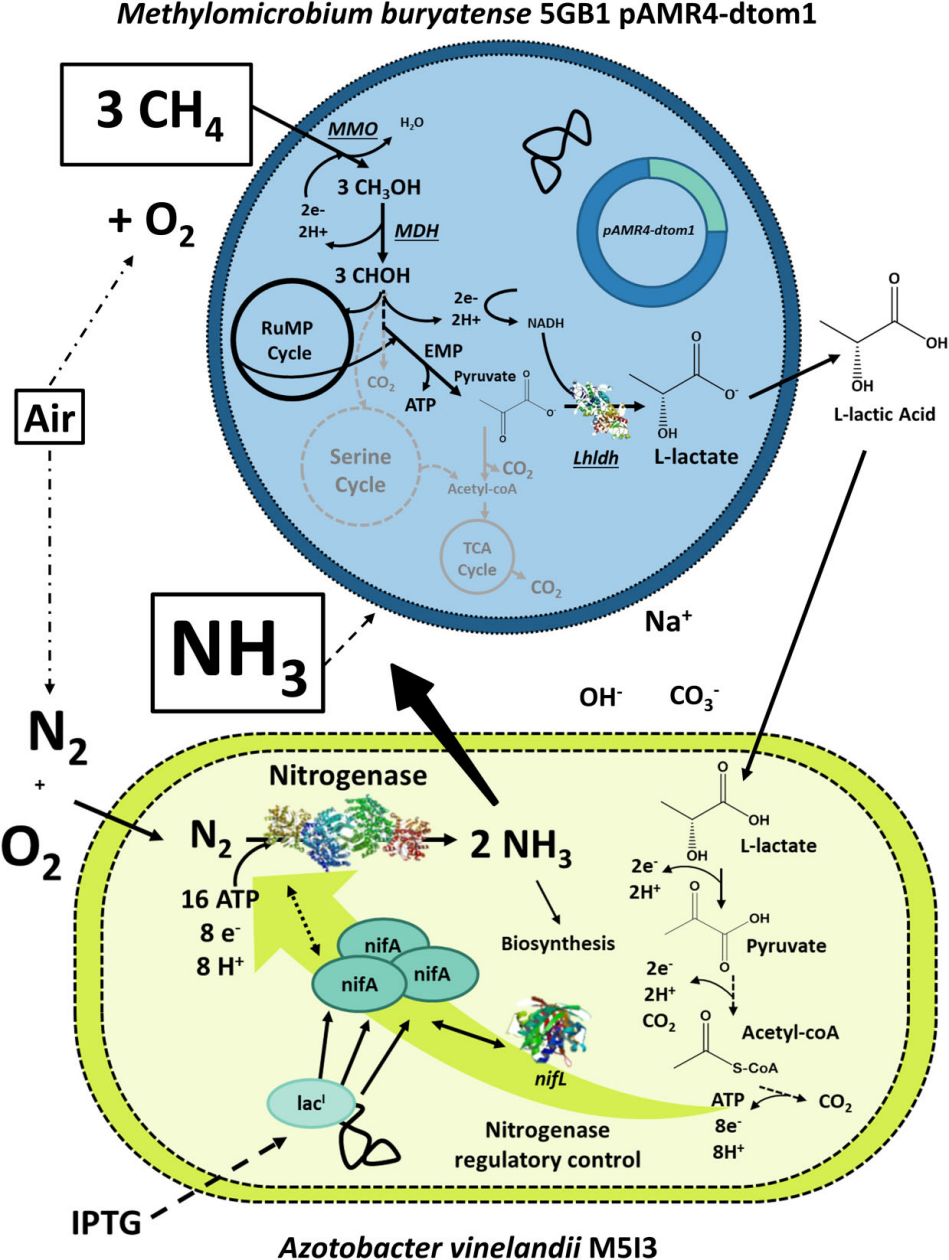
Conceptual design of a co-culture of engineered *A. vinelandii* and *M. buryatense* biologically using methane to power reduction of nitrogen in air to ammonia. Methanotroph *M. buryatense* 5GB1 converts methane (CH_4_) to glycolytic intermediate dihydroxyacetone phosphate (DHAP) through the action of methane monooxygenase (MMO: converts CH_4_ to methanol/CH_3_OH), methanol dehydrogenase (MDH: converts CH_3_OH to formaldehyde/CHOH), and the ribulose monophosphate (RuMP). DHAP enters the Embden–Meyerhof–Parnas pathway (EMP) where it is converted to pyruvate. This strain carries plasmid pAMR4-dtom1 with l-lactate dehydrogenase (Lhldh) from *Lactobacillus helveticus*, which converts pyruvate to l-lactic acid (Garg et al., 2018). Diazotroph *A. vinelandii* M5I3 consumes lactic acid and produces ATP and redox equivalents that drive the reduction of N_2_ in the air to NH_3_ using maximized expression of the enzyme nitrogenase. Nitrogenase gene expression is highly regulated by transcriptional repressor protein *nifL*, forming a complex with activator protein *nifA* under nonoptimal conditions. To improve NH_3_ production, chromosomal expression of *nifA* was manipulated by inserting the *lac* operon upstream of *nifA*, thus alleviating *nifL* regulatory control on nitrogenase activity in the presence of elevated concentrations of NH_3_ (Martinez-Argudo et al., 2004).

Conversion of CH_4_ to higher-value products represents an industrially feasible technology using efficient and genetically tractable methanotrophs, such as *M ethylomicrobium buryatense* (recently renamed *Methylotuvimicrobium buryatense* [Orata et al., 2018]), which activate CH_4_ by oxidation to methanol (CH_3_OH) (Kalyuzhnaya et al., 2015). Methane monooxygenase (MMO), the first enzyme in CH_4_ bioconversion, activates the relatively inert CH_4_ molecule and combines it with molecular oxygen from the air to produce CH_3_OH, further catalyzed to formaldehyde (CH_2_O) by methanol dehydrogenase (MDH) (Fig. 1) (Clomburg et al., 2017). Formaldehyde-derived carbon and redox carriers then integrate into central carbon metabolism through the Embden–Meyerhof–Parnas pathway, where metabolic engineering can prioritize biomanufacturing of organic acids such as l-lactate (C_3_H_6_O_3_) and muconic acid (C_6_H_6_O_4_) (Henard et al., 2016, 2019).

Meanwhile, nitrogen fixation to NH_3_ using the nitrogenase enzyme reduces atmospheric nitrogen (N_2_) to NH_3_ using up to 16 ATP and 8 reducing equivalents via the chemical reaction N_2_ + 8H^+^ + 16 ATP + 8e^−^ → 2 NH_3_ + H_2 _+ 16 ADP + 16 PO_4_^^−^^ (Dixon & Kahn, 2004). Since breaking the high-energy N_2_ triple bond (N≡N) is an energy-intensive activity, diazotrophs heavily regulate BNF activity to protect the organism from excess energy expenditure although the enzymatic process can be manipulated to increase production through regulatory system disruption (Fig. 1) (Brewin et al., 1999; Dixon & Kahn, 2004). While most diazotrophs require strictly anaerobic environments to protect oxygen-sensitive nitrogenase activity, a few have evolved to accommodate BNF in aerobic environments, including *Azotobacter vinelandii*, a model organism for aerobic BNF capable of using l-lactate as a feedstock (Dixon & Kahn, 2004).

In this report, we describe the development of synthetic co-culture for the microbial reduction of N_2_ to NH_3_ solely powered by using CH_4_ as a carbon source in a single vessel at ambient temperature and pressure. The proof-of-concept co-culture system consists of a methanotroph using CH_4_ to generate l-lactate and a diazotroph consuming l-lactate to power its transformation of atmospheric N_2_ into NH_3_.

## Materials and Methods

### Strains and Reagents

*Azotobacter vinelandii* strain DJ (“Deloriah Jacobs”) was obtained from the Dennis Dean laboratory at Virginia Tech (Setubal et al., 2009) and was the parent diazotrophic strain used in this work. *Methylomicrobium buryatense* (*Methylotuvimicrobium buryatense* [Orata et al., 2018]) strain 5GB1C and *Escherichia coli* strain S17-1 were obtained from the Mary Lidstrom laboratory at the University of Washington (Puri et al., 2015) and were the parent methanotrophic and transconjugation donor strains used, respectively. NEB DH5-α competent *E. coli* was obtained from New England BioLabs (Ipswich, MA, USA) and used for cloning. Oligonucleotide primers were purchased from Integrated DNA Technology (Coralville, IA, USA). Chemicals and reagents were purchased from Fisher Scientific (Pittsburgh, PA, USA), Sigma Aldrich, and VWR International (Radnor, PA, USA) unless otherwise specified. Gas sources in this study were purchased from Airgas (Radnor, PA, USA) unless specified.

### Strain Construction

*Methylomicrobium buryatense* 5GB1C was transformed with plasmid pAMR4-dtom1 (Garg et al., 2018) that expressed the fluorescent reporter gene dTomato1 (ex: 530 nm/em: 590 nm) downstream of the constitutive promoter P_mxaF_ using a triparental mating strategy with donor *E. coli* strain S17-1 (Puri et al., 2015). Plasmid insertion was verified by colony polymerase chain reaction (PCR). Cloning was performed in either NEB DH5-α competent *E. coli* cells (Ipswich, MA, USA) or *E. coli* Stellar cells (Clontech Laboratories, Inc., Mountain View, CA, USA).

Chromosomal genetic modifications in *A. vinelandii* followed protocols as previously detailed (Dos Santos, 2011). Plasmids using pGEM7f^+^ (Promega, Madison, WI, USA) as the backbone vector (Ortiz-Marquez et al., 2014) were constructed for chromosomal insertion using Gibson Assembly (Ipswich, MA, USA) following the manufacturer’s protocol and included 800-bp homologous fragments of the *nifL* and *nifA* genes amplified from *A. vinelandii* DJ genomic DNA by PCR using Phusion polymerase flanking inserted kanamycin resistance and *lac* operon genes. Transformations resulted in strains *A. vinelandii* M5I and M5T, containing an intact *nifL* fragment or a truncated *nifL* at position D392, respectively, upstream of an inducible *lac* promoter (p*tac*I) controlling *nifA* on the *A. vinelandii* DJ chromosome (Bali et al., 1992; Barney et al., 2017; de Boer et al., 1983). Chromosomal modifications were verified by colony PCR. Cloning was performed in either NEB DH5-α competent *E. coli* cells (Ipswich, MA, USA) or *E. coli* Stellar cells (Clontech Laboratories, Inc., Mountain View, CA, USA). Table 1 details all the strains used in this study.

**Table 1.** Microbial Strains and Plasmids Used in This Study

Species/Strains	Description	Source/Reference
*Azotobacter vinelandii*		
DJ	Wild type, Nif^+^	(Setubal et al., 2009)
M5T	DJ × pZT2; Nif^−^, Kan^R^	This study
M5I/M5I3	DJ × pZT3; Nif^+^, Kan^R^	This study
*Methylomicrobium buryatense*		
5GB1C	Wild type	(Puri et al., 2015)
*Escherichia coli*		
S17-1 λpir	Donor strain. Tpr Smr recA thi pro hsd(rm^+^) RP4-2-Tc::Mu::Km Tn7 λpir	(Puri et al., 2015)
Plasmids		
pGEM7f^+^		Promega
pCAH01	PtetA bla-tetR CoE1ori F1 oriV oriT trfA ahp	(Henard et al., 2016)
pAWP87	pAWP78 containing dTomato driven by *M. buryatense* mxaF promoter (PmxaF)	(Puri et al., 2015)
pAME	pAWP87 without dTomato	(Puri et al., 2015)
pAMR4	pAME harboring [RBS4]-Lhldh under the control of PmxaF	(Garg et al., 2018)
pAMR4-dtom1	pAME harboring [RBS4]-Lhldh under the control of PmxaF containing dTomato driven by *M. buryatense* mxaF promoter (PmxaF)	This study
pZT2	pGEM7f^+^-Truncated (D392) NifL-Kan-LacI-p*tac*I-NifA	This study
pZT3	pGEM7f^+^-Intact NifL-Kan-LacI-p*tac*I-NifA	This study

### Culture Media and Growth Conditions

Cells were cultured in modified Burk’s (B) media, nitrate mineral salts (NMS2) media, or a co-culture formulation (F) media, as detailed in Fig. 3 (Dos Santos, 2011; Puri et al., 2015). Stock solutions for all media included 10× B-C salts (B salts omitting carbon), 100× AvDJ phosphate buffer, 1 M carbonate buffer, 40 mM 5GB1 phosphate buffer solution, and 500× trace elements solution, filter-sterilized to prevent oxidation (Dos Santos, 2011; Puri et al., 2015). l-Lactate was added to 10× B-C salts and filter-sterilized during preparation, 10 g or as specified. Co-culture F media base consisted of 100-ml 1× B-salts, 10-ml 100× AvDJ phosphate buffer, 100-ml 10× NMS2, no NaCl base, except [–NMS2] where noted, 20-ml 40 mM 5GB1 phosphate buffer, 2-ml 500× trace elements, 745-ml sterile H_2_O, and 1.5% agar for plates. F25 medium contained 12.8-ml 5 M NaCl and 10-ml 1 M carbonate buffer. All media prepared regardless of mono/co-culture designation included 0.6-μg/ml kanamycin and 500 μM isopropylthio-β-galactoside (IPTG). Cultures were inoculated directly from glycerol stocks and grown in Hungate anaerobic culture tubes (Chemglass Life Sciences, Vineland, NJ, USA) sealed with a rubber stopper and crimped aluminum seal (Wheaton, Millville, NJ, USA) containing 2-ml liquid culture and 29-ml gaseous headspace unless noted. Tubes were incubated at a 45° angle in an NBS C24 benchtop incubator shaker (New Brunswick Scientific Co., Inc., Edison, NJ, USA) operating at 30 °C and 200 rpm. All sealed cultures were resupplied with 21% (v/v) CH_4_ in air every 24 hr except where noted.

Separate *A. vinelandii* and *M. buryatense* precultures were grown to exponential phase in native media until reaching an OD of ∼2.0, at which time preculture cells were pelleted, washed, and resuspended in 1-ml medium. Optical densities (OD_600_) were obtained on a Thermo Spectronic Genesys 20 (Thermo Scientific, Waltham, MA, USA). *Azotobacter vinelandii* and *M. buryatense* precultures were inoculated into fresh F medium as specified at OD_600_ 0.1 for preliminary assays. High-throughput screening was conducted to screen 13 different media formulations using a BioLectorI (M2P, GER) containing FlowerPlates (MTP-48-B, M2P, GER) covered with basic sealing foil (F-GP-10, M2P, GER HT). Cultures of *A. vinelandii* and *M. buryatense* were supplemented with 0.2% MeOH in place of CH_4_ and cultured in duplicate using 1-ml media at 30 °C with continuous shaking at 1,000 rpm. Biomass readings were taken every 15 min using the biomass optode sensor (620 nm) with a gain of 30.

For co-culture experiments, precultures were grown to exponential phase in the F25 medium. Preculture cells were pelleted, washed, and resuspended in 2-ml fresh medium to a final OD_600_ 0.05 for each species to achieve a 1:1 OD_600_ ratio unless otherwise noted. Sacrificial samples were collected at each time point. Gas was exchanged in remaining samples every 24 hr for the experiment duration. For ^15^N_2_ experiments, co-cultures were inoculated using a 10:1 *A. vinelandii:M. buryatense* ratio to a final OD_600_ 0.55. After sealing, Hungate tubes were flushed for 1 min using an air-alternative argon–oxygen gas mixture (21% O_2 _± 2%, residual Ar) (Airgas, Houston, TX, USA). Tubes were briefly vented to depressurize; 8 ml of either filtered air or dual-labeled ^15^N_2_ (98+ atom%, ICON/Berry & Associates, Inc., Dexter, MI. USA) gas was introduced using a needled syringe, and CH_4_ gas was added without gas removal. Gas was exchanged every 24 hr. At 72 hr, the system was supplemented with 0.5 ml of the initial media to maximize NH_3_ production for gas chromatography–mass spectrometry (GC-MS) analysis. Sacrificial samples were analyzed after 24 and 96 hr.

### Analytical Methods

Sacrificial samples were assayed for biomass (OD_600_), colony-forming units (CFU/ml), total lactate (g/L), and NH_3_ (mg/L). OD_600_ was used with cell dry weight (CDW) as an estimate of cell mass (for *A. vinelandii* DJ, 0.38-g CDW/OD/L; for *M. buryatense* 5GB1C, 0.24-g CDW/OD/L [Garg et al., 2018]). Culture viability was calculated as CFU/ml using the number of colonies that appeared when plated on each microbe’s respective native media. *Azotobacter vinelandii* M5I3 and co-culture samples were plated using sterile triplicate dilution on B medium plates supplemented with 0.6-μg/ml kanamycin and 500 μM IPTG and placed in a 30 °C stationary incubator for 7 days. *Methylomicrobium buryatense* 5GB1C pAMR4-dtom1 cultures were plated using sterile triplicate dilution on modified NMS2 medium supplemented with 50-μg/ml kanamycin and placed in a sealed chamber (Oxoid, Thermo Scientific, Waltham, MA, USA) containing 50% CH_4_-in-air and incubated at 30 °C for 7–10 days.

The supernatant was collected using centrifugation at 7,000 rpm for 6 min and analyzed for metabolite and substrate concentrations using ion-exclusion high-performance liquid chromatography on a Shimadzu Prominence SIL 20 system (Shimadzu Scientific Instruments, Inc., Columbia, MD, USA) equipped with an HPX-87H organic acid column (Bio-Rad, Hercules, CA, USA) and optimized operating conditions for peak separation (0.3-ml/min flow rate, 30 mM H_2_SO_4_ mobile phase, and column temperature 42 °C) (Cheong et al., 2016). High-performance liquid chromatography sample preparation involved the addition of 6-μl 6 N H_2_SO_4_, centrifugation at 13,000 rpm for 10 min, and filtration through an EMD Millipore 13-mm Nonsterile Millex^TM^ Syringe Filter (Waltham, MA, USA). Quantification of total NH_3_ was performed using an Ammonia Assay kit (Sigma Aldrich, St. Louis, MO, USA) and a Thermo BioMate spectrophotometer (Thermo Scientific, Waltham, MA, USA) at 340 nm.

## Results and Discussion

Figure 1 illustrates the conceptual design for a synthetic microbial co-culture using CH_4_ bioreforming to drive BNF and generate NH_3_ from atmospheric N_2_. Herein CH_4_ bioreforming is defined as the partial oxidation of CH_4_ at low temperatures and pressures by enzyme(s) or microorganism(s) resulting in liquid or gaseous products (in this study, l-lactate). The design employs a modular co-culture using a compartmentalized division of labor strategy to account for oxygen sensitivity of nitrogenase and maximize CH_4_ oxidation efficiency (Dixon & Kahn, 2004; Fei et al., 2014; Roell et al., 2019). In order to integrate cultures of *A. vinelandii* and *M. buryatense*, the CH_4_ bioreforming pathways needed to produce a feedstock capable of supporting NH_3_ production by *A. vinelandii*. l-Lactate was chosen as the extracellular energy transfer molecule because ethanol is toxic to *M. buryatense* (Garg et al., 2018), *A. vinelandii* NH_3_ production increased with increasing carbon chain length, and l-lactate represented a metabolically balanced CH_4_ bioreforming product. As conceived, with no carbon or nitrogen source other than CH_4_ and N_2_ provided, the system leveraged an obligately mutualistic metabolism, where *A. vinelandii* M5I3 depended on l-lactate generated from CH_4_ bioreforming by *M. buryatense* 5GB1C pAMR4-dtom1, which in turn relied on a portion of the NH_3_ produced by *A. vinelandii* M5I3 for its N source (Morris et al., 2013). In the proof-of-concept, N_2_ reduction by BNF used CH_4_ as the sole carbon and energy source but retained NO_3_ as a supplementary N source to initiate *M. buryatense* 5GB1C growth during system startup.

### Methane Bioreforming to l-Lactate Using *Methylomicrobium buryatense* 5GB1

The use of CH_4_ as a feedstock for chemical production and growth substrate requires carbon chain elongation and integration into central carbon metabolism, which the methanotrophic bacterium *M. buryatense* 5GB1C achieves through the ribulose monophosphate and the Embden–Meyerhof–Parnas pathways (Fig. 1). As a result, three molecules of CH_4_ are converted to pyruvate with the concomitant generation of one ATP and one NADH. Since the conversion of pyruvate to l-lactate consumes 1 NADH, the CH_4_-to-l-lactate pathway is redox and carbon balanced and ATP generating (Fig. 1). Therefore, in this study l-lactate acts as a carrier that minimizes carbon and energy losses. In addition, l-lactate exhibits easy cross-membrane transport, is an acceptable sole carbon and energy source by the co-culture partner *A. vinelandii*, and is tolerated by *M. buryatense* 5GB1 up to 1 g/L (Garg et al., 2018).

**Fig. 2.**
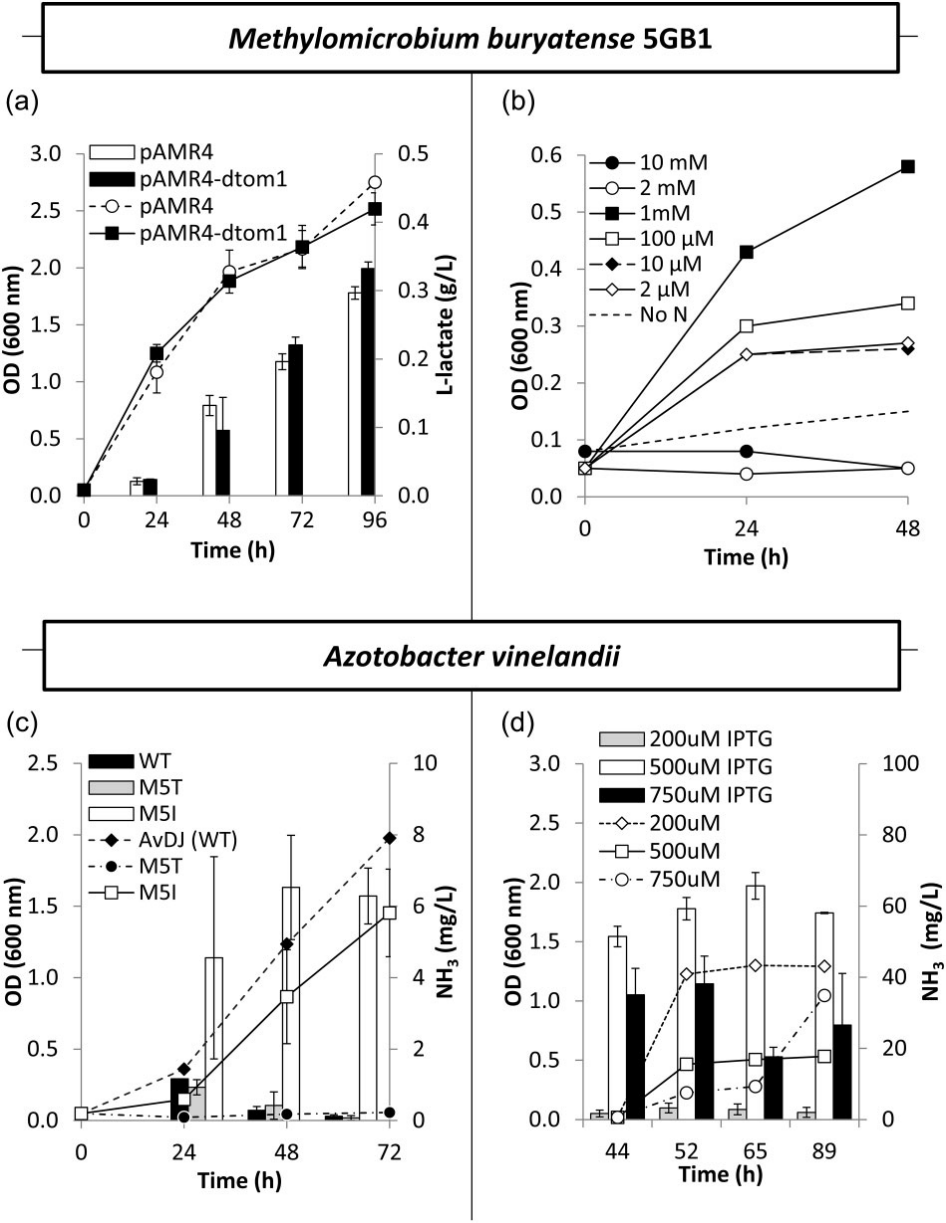
Engineered *M. buryatense* 5GB1C pAMR4-dtom1 and *A. vinelandii* M5I3 produce carbon and energy carrier l-lactate and reduce N_2_ to NH_3_, respectively. (a) *Methylomicrobium buryatense* 5GB1 pAMR4-dtom1 accumulated more than 0.3-g/L l-lactate in native NMS2 medium with daily gas exchange. (b) *Methylomicrobium buryatense* 5GB1C was most tolerant to AMS medium supplemented with 1 mM NH_4_Cl. (c) *Azotobacter vinelandii* M5I secreted increased NH_3_ at the cost of biomass accumulation when grown in Burk’s media containing 20-g/L glucose and 200 μM IPTG. Genetic modifications to M5T (D32-truncated repressor *nifL* and a *lac* operon inserted upstream of *nifA*) severely impacted overall performance. (d) 500 μM IPTG maximized NH_3_ production by *A. vinelandii* M5I3 in F22 medium supplemented with 10-g/L μ-lactate. Cell productivity was calculated using NH_3_ concentration per OD_600_. At 65 hr, cell productivity for *A. vinelandii* M5I3 with 200 μM IPTG was 2-mg NH_3_/L/OD, *A. vinelandii* M5I3 with 500 μM IPTG was 130-mg NH_3_/L/OD, and *A. vinelandii* M5I3 with 750 μM IPTG was 63-mg NH_3_/L/OD. Error bars represent standard deviation of three independent experiments.

*Methylomicrobium buryatense* 5GB1C has a relatively fast growth rate for a methanotroph, demonstrated genetic tractability, and observed tolerance to target molecules in this system (Gilman et al., 2015). Our previous efforts to maximize l-lactate production using heterologous expression of the *Lactobacillus helveticus* lactate dehydrogenase gene (*Lhldh*) in *M. buryatense* 5GB1C focused on evaluating native promoters and ribosomal binding site (RBS) sequences using a modular design approach (Garg et al., 2018). This work resulted in several strains of *M. buryatense* 5GB1C, including *M. buryatense* 5GB1C pAMR4, which produced more than 0.3-g/L l-lactate after 96 hr of cultivation in Hungate tubes (Garg et al., 2018). Additional efforts to construct a strain with fluorescent marker dTomato1 for co-culture applications resulted in strain *M. buryatense* 5GB1C pAMR4-dtom1 (Fig. 2a), which was used in subsequent experiments.

Additionally, since conflicting reports in the literature discuss inhibitory effects of NH_3_ on methanotroph activity under certain conditions (Bedard & Knowles, 1989; Nyerges et al., 2010), tolerance to NH_3_ accumulation and the potential for N-free growth were evaluated for *M. buryatense* 5GB1C; 1 mM NH_4_Cl tolerance was initially observed during growth at pH 9.5 when nitrate was replaced with varying concentrations of NH_3_ to make ammonia mineral salt (AMS) media (Fig. 2b). The ability of *M. buryatense* 5GB1C to grow on NH_4_Cl appeared to be pH-related, with strong NMS2 growth occurring around pH 9.5, while slower growth on AMS media occurred at lower pHs (Garg et al., 2018). However, attempts to transform an evolved variant of *M. buryatense* 5GB1C with plasmid pAMR4-dtom1, developed through directed evolution to tolerate up to 45 mM NH_4_Cl at pH 7.0, were not successful for unknown reasons, so further experiments used *M. buryatense* 5GB1C pAMR4-dtom1.

Furthermore, since *M. buryatense* 5GB1C possesses nitrite dissimilation genes reducing NO_3_ products to NH_3_ before assimilation, and additional analysis of the *M. buryatense* 5GB1 genome using NCBI BLAST identified BNF homologs suggesting potential native nitrogen fixation capability, using total NH_3_ as an analytical metric required knowledge of baseline NH_3_ secretion during native *M. buryatense* 5GB1C growth (Moreno-Vivian et al., 1989; Torre et al., 2015). Any NH_3_ production by *M. buryatense* 5GB1C grown in NMS2 media, however, was below the limit of detection for the assay (data not shown). Likewise, *M. buryatense* 5GB1C demonstrated no significant growth on N-free NMS2 medium under the conditions tested in this work (Fig. 2b).

### Conversion of Atmospheric Nitrogen to Ammonia Using *Azotobacter vinelandii*

Biological nitrogen fixation requires cellular energy input in the form of ATP and reducing equivalents to produce NH_3_ from N_2_ in the air using nitrogenases (Dixon & Kahn, 2004). The choice to use co-culturing methodology required using a feedstock compound capable of being produced by *M. buryatense* 5GB1. Early carbon-limited feedstock experimentation with wild-type *A. vinelandii* DJ demonstrated no observable growth on (C_1_) substrates but produced robust growth on ethanol (C_2_) and d- and l-lactic acid (C_3_) in liquid media (data not shown). Complete oxidation of l-lactate to CO_2_ by *A. vinelandii* results in the generation of six reducing equivalents per mole of l-lactate, which can produce up to 12 ATP when processed through the electron transport chain and oxidative phosphorylation (Igarashi & Seefeldt, 2003; Ledbetter et al., 2017; Poudel et al., 2018).

While the activity of nitrogenase activator *nifA* is negatively regulated under conditions of extracellular N excess, intracellular O_2_ presence, and carbon insufficiency to protect nitrogenase from inactivation and minimize excess energy expenditure, nitrogenase expression is manipulable using regulatory system disruption strategies although recent intensive efforts to sustain inducible NH_3_ performance for agricultural applications through the use of strong p*tac* promoters have indicated that manipulation of nitrogenase expression can cause strain instability and variability in observed NH_3_ yield (Ambrosio et al., 2024; Bali et al., 1992; Barney et al., 2015; Bennett et al., 2023; Brewin et al., 1999). Based on these reports, two regulatory disruption strategies were compared in this study to generate an NH_3_-secreting strain of *A. vinelandii*. These strategies included either an intact nitrogenase transcriptional repressor *nifL* fragment or a truncated *nifL* at position D392 in the C-terminal domain, labeled as *A. vinelandii* M5I and M5T, respectively, and chromosomal insertion of a *lac*-controlled promoter (p*tac*I) immediately upstream of *nifA* within the *nifLA* operon on the *A. vinelandii* DJ chromosome (Bali et al., 1992; Brewin et al., 1999). Since σ^54^-dependent nitrogenase activator protein *nifA* is negatively regulated through complex formation with nitrogenase transcriptional repressor *nifL*, disrupting the *nifL***-***nifA* complex formation by overexpressing *nifA* results in continuous nitrogenase activity even in the presence of elevated NH_3_ concentration (Dixon & Kahn, 2004; Martinez-Argudo et al., 2004).

The intact *nifL* M5I variant demonstrated a fivefold increase in NH_3_ over the wild-type and truncated (M5T) *nifL* strains (Fig. 2c) when grown on N-free B medium containing 20-g/L sucrose and 200 μM IPTG. Overall, M5I culture growth matched reported trends for slight biomass decrease with increased NH_3_ secretion (Ambrosio et al., 2017). However, the truncated *nifL* strategy (M5T) resulted in both poor growth and low NH_3_ secretion, suggesting that disruption of the C-terminal domain of *nifL* at D392 detrimentally altered the strain profile (Fig. 2c). Notably, while the M5I strain outperformed both the wild-type and M5T *A. vinelandii* strains, the cultures exhibited some variability in measured extracellular NH_3_, similar to other reports (Ambrosio et al., 2024; Barney et al., 2015; Bennett et al., 2023; Ortiz-Marquez et al., 2014). In an effort to understand the extent of the variability, three rounds of subculturing of multiple technical replicates from multiple glycerol stocks indicated that initial NH_3_ yield differences between stocks and technical replicates observed during the first round carried through subsequent rounds of subculturing. However, populations with substantially increased NH_3_-producing capability observed in the initial round of subculturing continued to retain relatively higher NH_3_ yield compared to the lower-performing populations in subsequent rounds, suggesting that while the original stock culture population likely consisted of a mix of producer and nonproducer cells, it is possible to achieve a population with elevated NH_3_ yield compared to the wild-type strain through subculturing (data not shown). The best-performing strain, *A. vinelandii* M5I3, was used for further studies. IPTG optimization of *A. vinelandii* M5I3 behavior at 500 μM resulted in a 10-fold total increase in extracellular NH_3_ accumulation when using 20-g/L glucose as the carbon source (Fig. 2d). The highest and most stable NH_3_-secreting induction concentration, 500 μM IPTG, was used for future experiments.

### Design of Medium and Conditions Supporting Co-cultivation

**Fig. 3.**
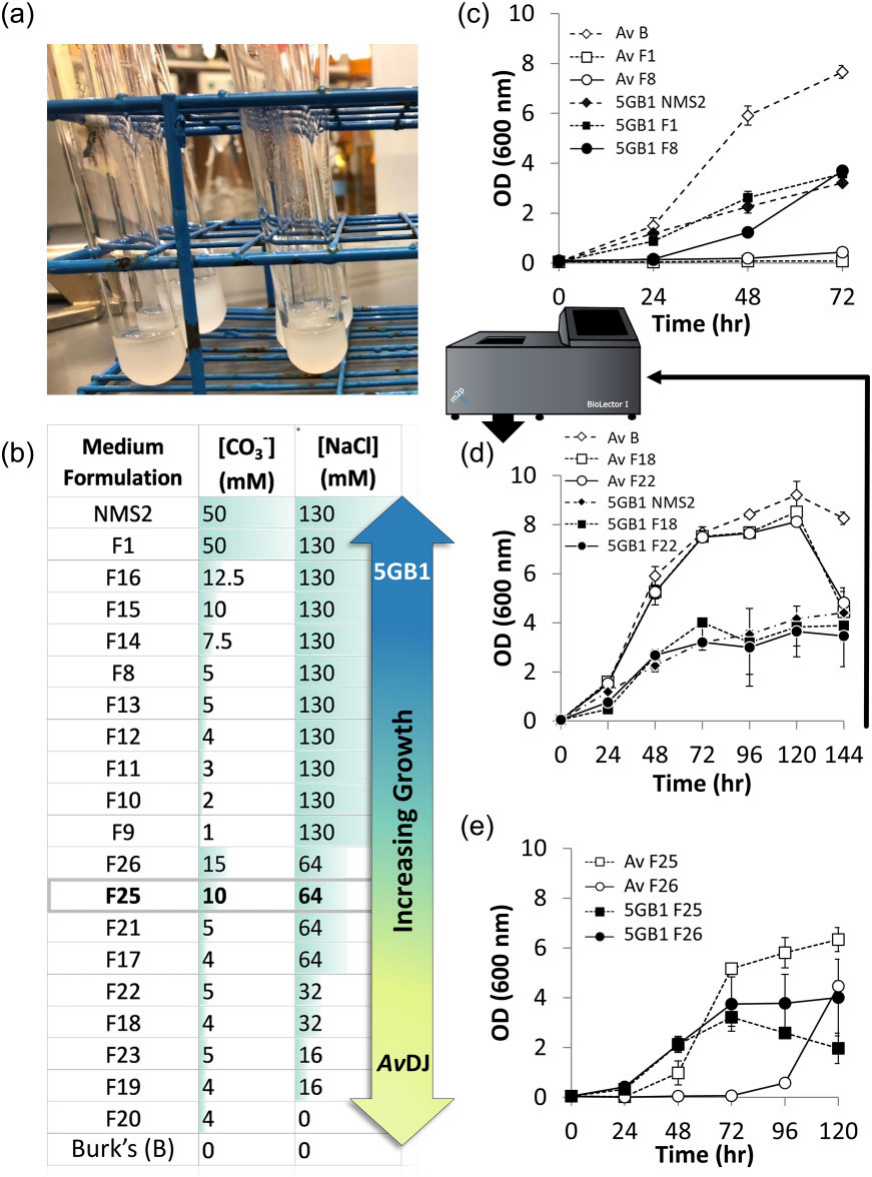
Development of a co-culture system supporting growth of *M. buryatense* and *A. vinelandii*. (a) Cultures shown in Hungate tubes. (b) Twenty-seven iterations (20 shown) of F media with various carbonate (CO_3_^2−^) and NaCl concentrations. (c) Early iterations did not adequately support the growth of both organisms. (d) High-throughput screening using the BioLector and media supplemented with 0.2% (v/v) methanol in place of CH_4_ indicated F18 and F22 supported biomass yields closest to those for wild-type strains in their native media. (e) F25 maximized exponential growth of each strain in Hungate tubes when methanol was replaced with 21% (v/v) CH_4_ in air.

The use of a co-culture strategy required establishing an environment capable of supporting the growth of both organisms (Fig. 3a). *Methylomicrobium buryatense* 5GB1C and *A. vinelandii* were isolated from different habitats, and a comparison of their laboratory bench growth medias identified substantial differences in pH and salinity. Haloalkaliphilic *M. buryatense* 5GB1C preferentially grows in NMS2 media containing sodium (Na) concentrations above 500 mM and an alkaline pH above 9.0 (Zhao et al., 2014). Conversely, mesophilic soil bacterium *A. vinelandii* prefers neutral pH and additionally contains the enzyme nitrogenase inhibited above pH 8.5 (Bali et al., 1992; Dos Santos, 2011; NCBI, 2018). Despite these differences, both *M. buryatense* 5GB1C and *A. vinelandii* DJ grow optimally at 30 °C, and the inclusion of 21% (v/v) CH_4_ in the *A. vinelandii* culture environment produced no discernible impact on biomass formation (data not shown). Selective pressure markers and induction reagents for gene expression also had to be mutually compatible, including tolerance to induction agent carrier solvent (Garg et al., 2018).

Twenty-seven iterations of dual monoculturing converged on a mutually compatible medium. Starting with a 1:1 ratio of B-glucose medium (10-g/L glucose) to NMS2 medium, CO_3_^2^−^^ and NaCl were varied as detailed in Fig. 3b. *Azotobacter vinelandii* DJ tolerated up to 7.5 mM carbonate after a lag phase of ∼50 hr, but higher concentrations were inhibitory to its growth (Fig. 3c). Additionally, increasing NaCl concentration increased the lag phase in *A. vinelandii* cultures. While temporarily replacing CH_4_ with 0.2% MeOH, an acceptable alternate *M. buryatense* 5GB1C substrate, to facilitate operational efficiency, a series of media formulations were iteratively evaluated using the rate of biomass formation as the performance metric (Fig. 3b and d). Two media formulations, F18 and F22, were chosen for further studies in Hungate tubes because both organisms showed a growth profile similar to those seen in their native media (Fig. 3d). Finally, since media formulation work through F22 used 10-g/L glucose as the *A. vinelandii* carbon source, glucose was replaced with representative anticipated co-culture-generated substrate l-lactate to generate medium formulation F25. Monocultures of *M. buryatense* 5GB1 pAMR4-dtom1 and *A. vinelandii* M5I3 cultured in F25 medium containing either 21% (v/v) CH_4_ refreshed every 24 hr or 10-g/L l-lactate, respectively, achieved growth rates comparable to those on their native media (Fig. 3e).

### Production of Ammonia From Atmospheric Nitrogen in Methane-Fed Co-culture

**Fig. 4.**
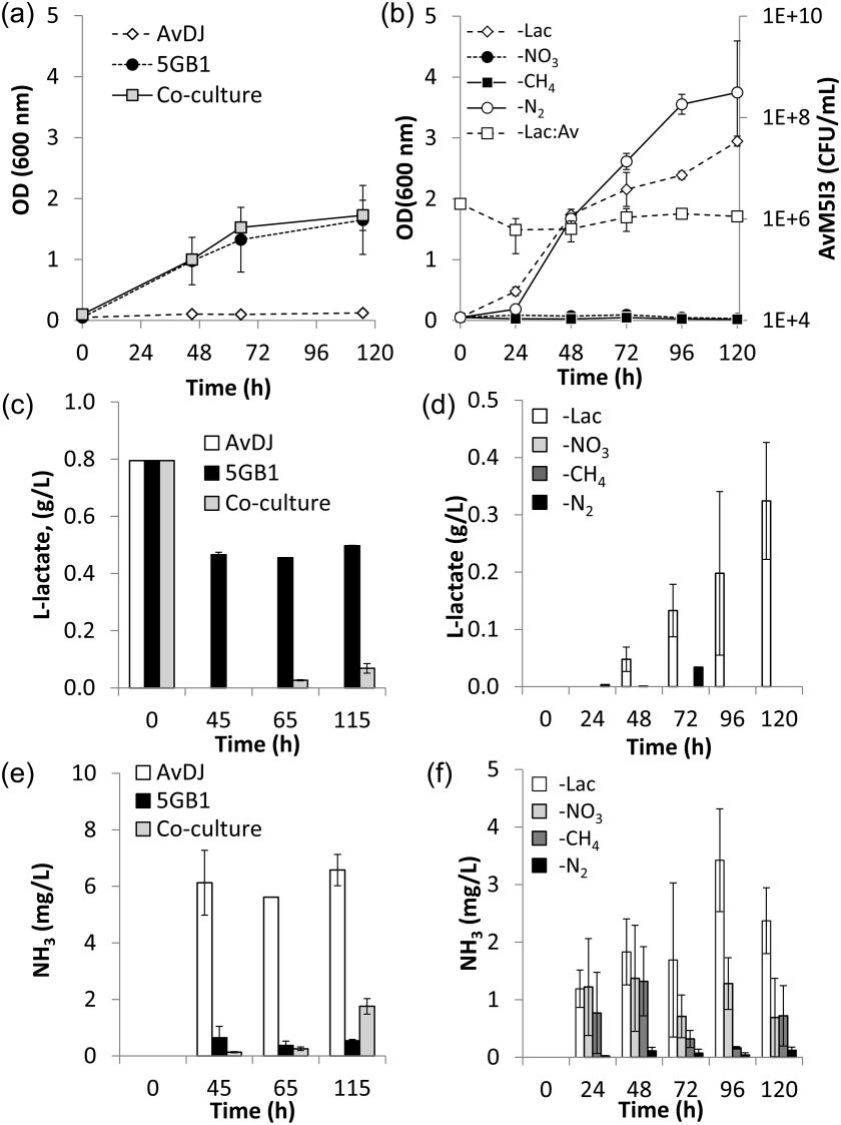
CH_4_-powered NH_3_ production by co-culture system in modified F25 medium. *Methylomicrobium buryatense* 5GB1C pAMR4-dtom1 and *A. vinelandii* M5I3 were combined using a 1:1 OD_600_ ratio and cultured in F25 medium. (a, c, and e) Mono- and co-cultures were able to grow and produce lactate and NH_3_ when supplementing F25 medium with 1-g/L Na l-lactate and 10 mM NO_3_. Left: (a) Optical density (OD_600_), (c) total lactate (g/L), and (e) total NH_3_ (mg/L). (b, d, and f) Omitting nutrients impacted both biomass and NH_3_ production. *Azotobacter vinelandii* CFU/ml at time = 0 hr estimated using Myers et al. (2013). Right: (b) OD_600_ and cell viability (CFU/ml), (d) total l-lactate (g/L), and (f) total NH_3_ (mg/L). Biological triplicates were assayed in parallel using a sacrificial sampling method.

A modified F25 medium containing 1-g/L l-lactate and 10 mM NO_3_, with the initial pH reduced to 7.5, became the basis for co-culture experiments to accommodate strain sensitivities. Monocultures were co-cultivated to performance initially, and subsequent experiments tracked organism viability using the CFU/ml counting method. Biomass (OD), total lactate (g/L), and extracellular aqueous NH_3_ (mg/L) were used as metrics of system behavior (Fig. 4). Biomass increase in most cultures demonstrated the strains survived both alone and together under the modified environmental conditions. Accumulation of l-lactate and NH_3_, respectively, in the monocultures suggested that the strains retained their modified traits; however, total accumulation was lower in the co-culture than in the parallel monocultures (Fig. 4c and e), which could be due to several factors, including competition for limiting resources, metabolite cross-consumption by organisms within the culture, or environmental stress on the system.

Further evaluation of the defined minimal medium system explored the omission of l-lactate, as well as omitting l-lactate and NO_3_, CH_4_, and N_2_, to represent the CH_4_-only [minus Lac], N_2_-only [minus NO_3_], all carbon-omitted [minus CH_4_], and N_2_-omitted [minus N_2_], conditions, respectively. [Minus l-Lac] cultures demonstrated a substantial increase in all metrics for system performance, resulting in a more than fourfold increase in lactate accumulation to 0.33-g/L l-lactate and a twofold increase in NH_3_ accumulation to 3.42-mg/L NH_3_, as well as substantial biomass accumulation (Fig. 4b, d, and f). Specifically, NH_3_ accumulation in the [minus Lac] culture outpaced accumulation in all other co-cultures, especially after 72 hr when the culture appeared to shift into the stationary phase in a similar accumulation pattern to those observed for preliminary monocultures of *A. vinelandii* M5I3. Given the known inhibitory effect of lactate on *M. buryatense* 5GB1, supplementing early experiments with 1.0-g/L lactate likely limited initial system performance (Henard et al., 2016). *Azotobacter vinelandii* M5I3 viability (CFU/ml) also remained relatively constant throughout the experiment, suggesting that CH_4_-derived carbon is capable of sustaining *A. vinelandii* M5I3 in the system.

Cultures omitting NO_3_ [minus NO_3_] and all carbon [minus CH_4_], respectively, saw neither substantial biomass accumulation nor any accumulation of l-lactate in either condition, although both conditions did report similar accumulations of up to 1-mg/L NH_3_ (Fig. 4b, d ,and e). Since these components are critical for *M. buryatense* 5GB1 growth in the F25 medium, they also impact its ability to produce carbon substrate, limiting the *A. vinelandii* carbon source. However, *M. buryatense* 5GB1 is adaptable to the AMS medium, suggesting that iterative optimization might increase performance under the [minus NO_3_] condition. A small amount of NH_3_ appeared in the both the [minus NO_3_] and [minus CH_4_] cultures. However, the stationary NH_3_ concentration in the sacrificial samples over time suggests that preliminary activity by the cells was not sustainable without the carbon transformation observed in the more supported [minus lac] condition.

The condition omitting atmospheric nitrogen [minus N_2_], but retaining NO_3_, was also evaluated to determine the extent to which atmospheric N_2_ reduction by nitrogenase was responsible for NH_3_ accumulation in the system, since NH_3_ accumulation could also theoretically result from amino acid degradation. For the [minus N_2_] condition, cultures were flushed with a 21% O_2_–balance argon gas mixture to eliminate atmospheric nitrogen while retaining O_2_ essential for CH_4_-activating MMO enzyme activity (Kalyuzhnaya et al., 2015). Despite overall biomass generation exceeding the [minus Lac] culture, observed NH_3_ accumulation was three orders of magnitude lower than observed for the [minus Lac] condition, strongly suggesting that extracellular NH_3_ accumulation requires atmospheric N_2_.

## Conclusions

Remote renewable CH_4_ represents a distributed high-energy feedstock with the potential to serve as a substrate for bioproduction. In this report, a microbial co-culture system utilized CH_4_ bioreforming to power BNF at ambient pressure and temperature in a single vessel using only CH_4_ and air (N_2_ and O_2_). pH and salinity were the primary factors affecting the development of a medium supporting the co-cultivation of both organisms with carbon and nitrogen availability heavily influencing NH_3_ production.

E-supplementary data of this work can be found in the online version of the paper.

## Supplementary Material

kuae044_Supplemental_File

## Data Availability

The data underlying this article are available in the article and in its online supplementary material.
