# Supplementary material for: A synthetic co-culture for bioproduction of ammonia from methane and air
Source: J Ind Microbiol Biotechnol. 2024 Nov 18;51:kuae044. doi: 10.1093/jimb/kuae044 (PMC11653078; doi:10.1093/jimb/kuae044)
Supplement: kuae044_Supplemental_File [file kuae044_supplemental_file.pdf]

Supplementary Materials
for

A synthetic co-culture for bioproduction of ammonia from methane and air

Anna Morgan Crumbley[1,4*], Shivani Garg[1,3], Jonathan Ling Pan[1], and Ramon Gonzalez[1, 2]

[1]Department of Chemical and Biomolecular Engineering, Rice University, Houston, TX, USA, 77251

[2]Current address: Department of Chemical, Biological and Materials Engineering, University of South Florida, Tampa, FL, USA, 33620

[3]Current address: 8045 Lamon Avenue, LanzaTech, Skokie, IL, 60077

[4]Current address: U.S. Army DEVCOM Chemical Biological Center (DEVCOM CBC), 8908 Guard Street, E3831, Gunpowder, MD, 21010

*Corresponding author. Email: anna.m.crumbley2.civ@army.mil / Phone: (410)-417-0536

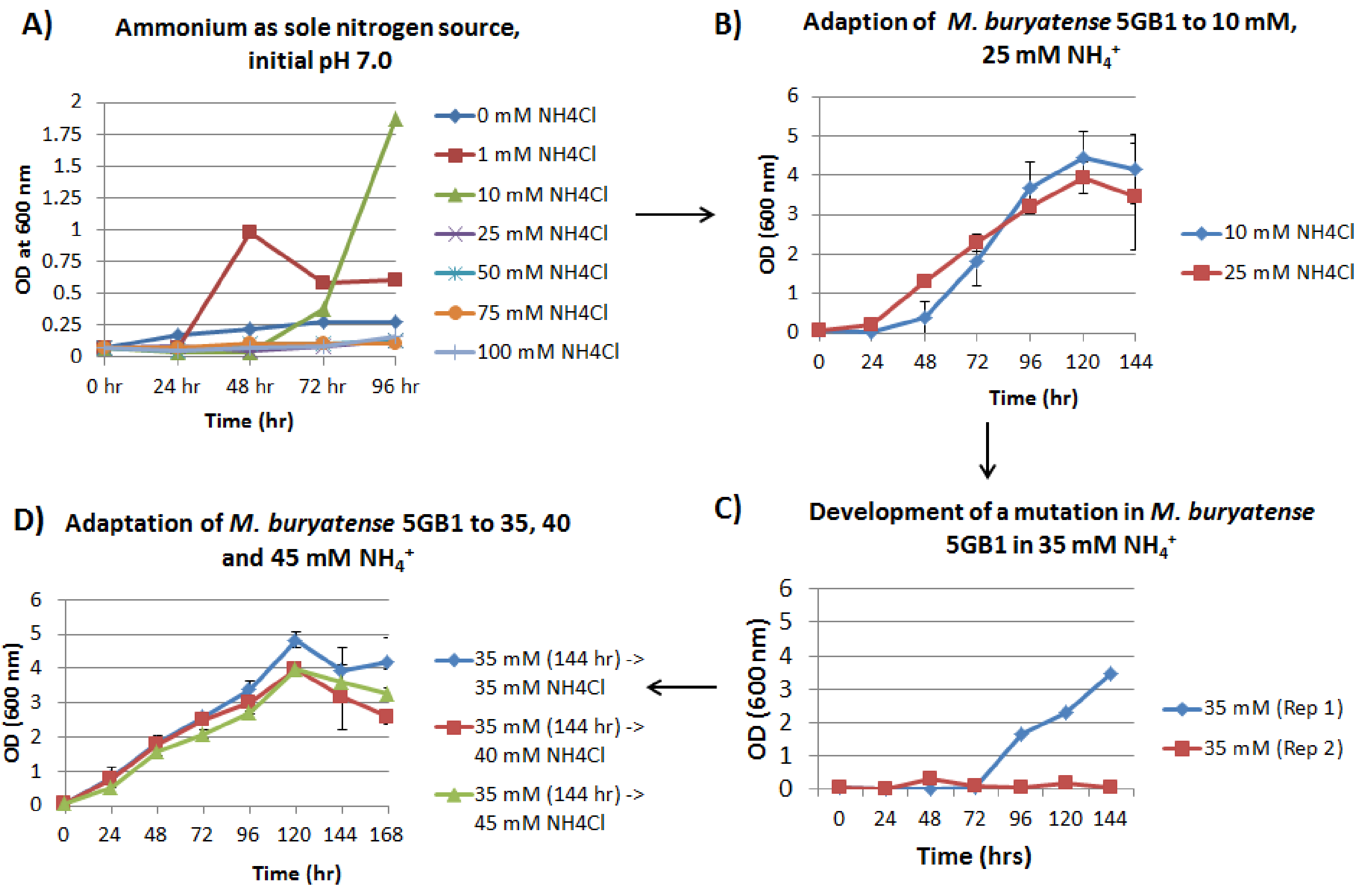

A)
Ammonium as sole nitrogen source, initial pH 7.0
OD at 600 nm
2
1.75
1.5
1.25
1
0.75
0.5
0.25
0
0 hr
24 hr
48 hr
72 hr
96 hr
Time (hr)
0 mM NH4Cl
1 mM NH4Cl
10 mM NH4Cl
25 mM NH4Cl
50 mM NH4Cl
75 mM NH4Cl
100 mM NH4Cl
B)
Adaption of M. buryatense 5GB1 to 10 mM, 25 mM NH4+
OD (600 nm)
6
5
4
3
2
1
0
0
24
48
72
96
120
144
Time (hr)
10 mM NH4Cl
25 mM NH4Cl
C)
Development of a mutation in M. buryatense 5GB1 in 35 mM NH4+
OD (600 nm)
6
5
4
3
2
1
0
0
24
48
72
96
120
144
Time (hrs)
35 mM (Rep 1)
35 mM (Rep 2)
D)
Adaptation of M. buryatense 5GB1 to 35, 40 and 45 mM NH4+
OD (600 nm)
6
5
4
3
2
1
0
0
24
48
72
96
120
144
168
Time (hr)
35 mM (144 hr) -> 35 mM NH4Cl
35 mM (144 hr) -> 40 mM NH4Cl
35 mM (144 hr) -> 45 mM NH4Cl
